# β4 and β6 Integrin Expression Is Associated with the Subclassification and Clinicopathological Features of Intrahepatic Cholangiocarcinoma

**DOI:** 10.3390/ijms19041004

**Published:** 2018-03-27

**Authors:** Yurie Soejima, Miho Takeuchi, Takumi Akashi, Motoji Sawabe, Toshio Fukusato

**Affiliations:** 1 Department of Molecular Pathology, Graduate School of Health Care Sciences, Tokyo Medical and Dental University, Tokyo 113-8510, Japan; soejima.mp@tmd.ac.jp (Y.S.); apmi56230@gmail.com (M.T.); m.sawabe.mp@tmd.ac.jp (M.S.); 2 General Medical Education and Research Center, Teikyo University, Tokyo 173-8605, Japan; 3 Department of Diagnostic Pathology, Graduate School of Medical and Dental Sciences, Tokyo Medical and Dental University, Tokyo 113-8510, Japan; akashi.path@tmd.ac.jp

**Keywords:** intrahepatic cholangiocarcinoma, integrin, classifications, tenascin, laminin, TGF-β1

## Abstract

Intrahepatic cholangiocarcinoma (ICC) is a heterogeneous group of cancers of the intrahepatic biliary tract. However, few studies have evaluated integrin expression according to an ICC subgroup. We immunohistochemically investigated α6β4 (β4) and αvβ6 (β6) integrin expressions in 48 ICCs, and evaluated their relationship with clinical and pathological parameters and ligand expression, as well as transforming growth factor (TGF)-β1. β4 and β6 expressions were detected in 46 (96%) and 35 (73%) ICC cases, respectively. We classified ICC into negative, low (β4, 29 cases; β6, 36 cases), or high (β4, 19 cases; β6, 12 cases) integrin expression groups. β4 and β6 integrin levels were higher in the non-peripheral central localization type ICC than in the peripheral localization type; they were also higher in the periductal-infiltrating or intraductal-growth types than in the mass-forming type ICC; lastly, they were higher in the well-differentiated type than in the poorly-differentiated type ICC. High expression was related to bile duct invasion. In addition, β4 and β6 expressions were associated with mucin production and the expression of cytoplasmic epithelial membrane antigen, laminin-5, and tenascin-C. TGF-β1 was correlated with β6 expression and poor overall survival. These results suggest that integrin expression is associated with subclassification and clinicopathological features of ICC through the coincident expression of their ligands and TGF-β1.

## 1. Introduction

Intrahepatic cholangiocarcinoma (ICC) is the second most common cancer arising in the liver; it comprises 15% of primary liver cancers. Surgical resection is the only potentially curative treatment in patients with ICC, but its resectability remains low (15% at the time of diagnosis), and the outcome of patients after resection has hardly improved, with a median survival time of fewer than three years [1,2]. ICC is defined as cholangiocarcinoma that is located proximally in either the first-order or higher peripheral branches of the right and left hepatic bile duct, which is distinguished from perihilar and distal extrahepatic cholangiocarcinoma [3]. The incidence of ICC has increased in the past three decades, while the incidence of perihilar and distal extrahepatic cholangiocarcinoma has remained stable [2,4].

ICC is a heterogeneous group of cancers of the intrahepatic biliary tract. Several ICC subclassifications have been proposed based on localization, disease extent, gross morphology, histology, biology, and cellular origin, as well as molecular classification [5]. According to anatomical localization, the large duct type of ICC is located in the first to third-order branches of the right and left hepatic bile ducts, which contain the peribiliary glands, while the peripheral small duct type involves the septal and interlobular bile ducts without the peribiliary glands [6]. Macroscopically, ICC is classified into mass-forming (MF), periductal-infiltrating (PI), intraductal-growth (IG), and mixed MF and PI (MF+PI) types [7,8]. Komuta et al. classified ICCs into muc-ICCs with mucin-production, and mixed ICCs with focal hepatocytic differentiation and ductular features, according to their similarity to the various cholangiocyte phenotypes of different site origins in the biliary tree [9]. Liau et al. divided ICCs into the bile duct and cholangiolar subtypes based on cholangiocytic differentiation and coincident mutational analysis [10]. Hayashi et al. classified ICCs into Type 1 or Type 2 based on mucin productivity and immunophenotypes, and showed their relationships to genetic changes and clinicopathological significance [11]. However, these subclassifications require refinement to provide useful information for the diagnosis and treatment of ICC. 

Integrins are cell surface receptors that connect the cytoskeleton to the extracellular matrix (ECM) and regulate cell adhesion and movement. Integrins comprise a family of transmembrane glycoproteins composed of α and β subunits, forming 24 αβ heterodimeric members that are widely distributed and interact with many ligands, including tenascin and laminin. Integrins are known to be important at every stage of cancer, including tumor cell migration, invasion, proliferation, and survival; they also contribute to tumor progression and metastasis [12].

The α6β4 (β4) integrin is a biliary-type integrin that is expressed on normal and proliferating biliary epithelia and cholangiocarcinomas, but not on hepatocytes and hepatocellular carcinoma (HCC) [13]. β4 integrin, which is a receptor for laminin, may also play key roles in tumor cell invasion and tumor development. The high expression of α6β4 integrin has recently been associated with bile duct invasion and lymph node metastasis, as well as overall survival (OS) in patients with ICC [14], but the relationship between β4 expression and ICC subclassification has not yet been fully described. 

The αvβ6 (β6) integrin is a receptor for the extracellular matrix proteins, including fibronectin, vitronectin, and tenascin. It is expressed exclusively on epithelial cells, and typically only during tissue remodeling, which occurs in inflammation and cancer. However, β6 is not expressed in normal adult epithelia [15]. β6 is reportedly up-regulated in cholangiocarcinoma, and is a highly specific immunohistochemical marker for cholangiocarcinoma [16]. In addition, β6 integrin serves as an immunohistochemical marker for lymph node metastasis and promotes the invasion in cholangiocarcinoma [17]; however, the β6 integrin expression status in subgroups of ICC remains unknown. 

In our previous study, we evaluated the expressions of the αvβ6, α6β4, and α3β1 integrins and their ligands, fibronectin and laminin, in cholangiolocellular carcinoma (CLC), cholangiocarcinoma, and HCC. Our results revealed the significant down-regulation of the αvβ6, α6β4, and α3β1 integrins in CLC in contrast to their high expressions in cholangiocarcinoma, as well as the distinct immunolocalization of fibronectin and laminin in CLC [18]. These results suggest that integrin expression may be of diagnostic value and a useful tool for defining the clinical and pathological entities of CLC and cholangiocarcinoma.

However, few studies have reported the expression of these integrins and ligands in ICC subgroups. The present study immunohistochemically investigated the expressions of β4 and β6 integrins and their ligands, laminin-5 and tenascin-C, in 48 ICCs, and evaluated their relationships with clinical and pathological parameters. Furthermore, we investigated the transforming growth factor (TGF)-β1, which is known to be activated by the β6 integrin and induce cancer-associated myofibroblasts that are positive for α-smooth muscle actin (SMA) [19,20]. The results suggested that integrin expression is associated with the subclassification and clinicopathological features of ICC through their ligands, laminin-5 and tenascin-C, and TGF-β1.

## 2. Results

### 2.1. Relationships between β4 and β6 Integrin Expressions and Clinicopathological Features in Patients with Intrahepatic Cholangiocarcinoma (ICC)

**Figure 1:**
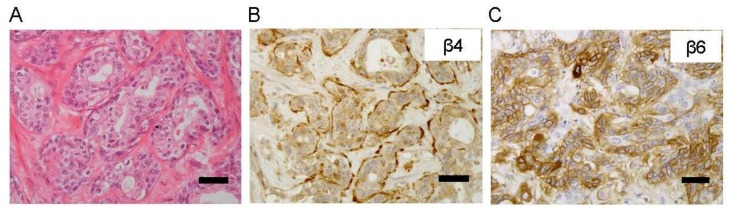
Expressions of β4 and β6 integrin in intrahepatic cholangiocarcinoma. Hematoxylin and eosin staining (**A**). The expressions of the β4 and β6 integrins are shown in the basal lamina (**B**), cell membranes, and cytoplasm (**C**). Scale bar, 50 μm. Original magnification, ×200.

Positive immunohistochemical staining for β4 and β6 integrins were observed in 46 (96%) and 35 (73%) of 48 ICCs, respectively. The localization of β4 and β6 expressions were classified as cell membrane, basal lamina, or cytoplasmic patterns (Figure 1A–C) [18]. In some cases, mixed expression patterns were observed simultaneously. β4 and β6 expression levels were classified into negative to low (29 and 36 cases; 60.4% and 75%, respectively) and high (19 and 12 cases; 39.6% and 25%, respectively) groups. 

**Figure 2:**
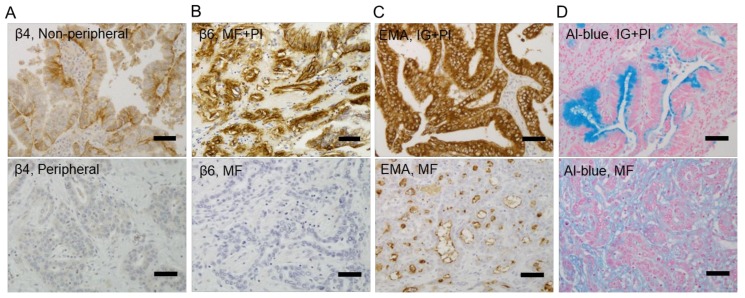
The expressions of β4 and β6 integrin in association with the localization and macroscopic types of intrahepatic cholangiocarcinoma. β4 and β6 integrin expression levels are higher in the non-peripheral central localization type than in the peripheral localization type intrahepatic cholangiocarcinoma (ICC) (**A**), and higher in the intraductal-growth and periductal-infiltrating types (IG+PI) than in the mass-forming type (MF) intrahepatic cholangiocarcinoma (**B**). A cytoplasmic epithelial membrane antigen (EMA)-positive pattern and Alcian blue (Al-blue)-positive intracytoplasmic dense mucus staining were observed in the high-integrin expression groups, including IG+PI, while a luminal EMA positive pattern and faint Alcian blue-positive mucus staining were evident in the low-integrin expression groups, including the MF group (**C**,**D**). Scale bar, 50 μm. Original magnification, ×200.

The correlations between β4 and β6 integrin expressions and the localization, macroscopic type, growth type, bile duct invasion, and differentiation of ICC were statistically significant (Table 1). β4 and β6 integrin expression levels were higher in the non-peripheral central localization type ICC than in the peripheral localization type (*p* = 0.033 and 0.0092, respectively) (Figure 2A); higher in the periductal-infiltrating or intraductal-growth types than in the mass-forming type (*p* = 0.0022 and 0.028, respectively) (Figure 2B); higher in the infiltrative-growth type than in the expansive-growth types (*p* = 0.015 and 0.065, respectively), and lower in the poorly-differentiated type than in the well-differentiated types (*p* = 0.023 and 0.0068, respectively). High expression was related to bile duct invasion (*p* = 0.0053 and 0.0026, respectively). β4 and β6 integrin expressions were both high in 10 cases; β4 only was high in nine cases, β6 only was high in two cases, and there was a negative to low expression of both in 27 cases. β4 or β6 integrin expressions were not significantly correlated with overall survival (*p* = 0.42 and 0.47, respectively, Supplemental Figure S1).

Classification of the β6 expression levels as negative or positive revealed a significantly high incidence of lymph node metastasis in the positive expression group (*p* = 0.045) (Supplemental Table S1). 

### 2.2. Correlations between β4 and β6 Integrin Expressions and EMA-Positive Patterns or Mucus Production in ICC

The expression patterns of epithelial membrane antigen (EMA) in normal bile ducts are closely related to duct size. Smaller bile ducts show a luminal membranous pattern, while larger ducts show a cytoplasmic pattern [21]. In the ICC cases, 37 (77%) and 11 (23%) of the cases showed predominantly cytoplasmic and luminal patterns, respectively. The expressions of β4 and β6 were stronger in the ICCs with cytoplasmic EMA-positive patterns than in those with luminal EMA-positive patterns (*p* = 0.018 and 0.16, respectively) (Table 1, Figure 2C). Alcian blue-positive mucus staining was more frequently detected in the cases with high β4 or β6 integrin expression than in the cases with low expression (*p* = 0.011 and 0.018, respectively) (Table 1, Figure 2D). 

### 2.3. Relationships between β4 and β6 Integrin Expressions and Laminin-5 or Tenascin-C Expressions in ICC 

**Figure 3:**
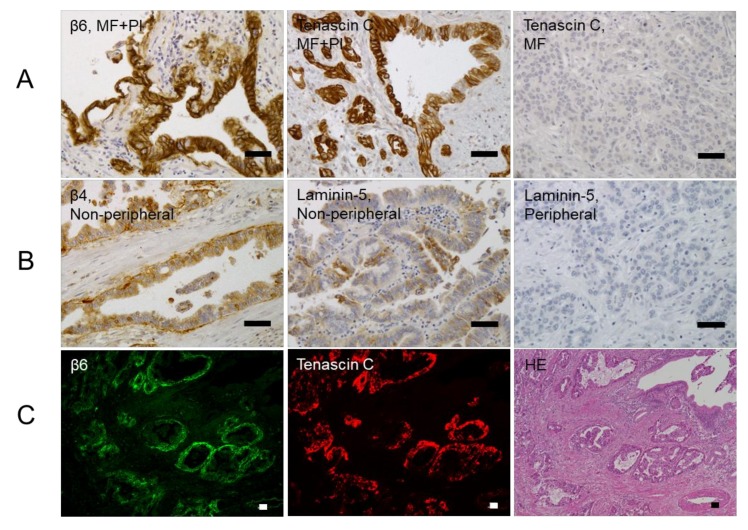

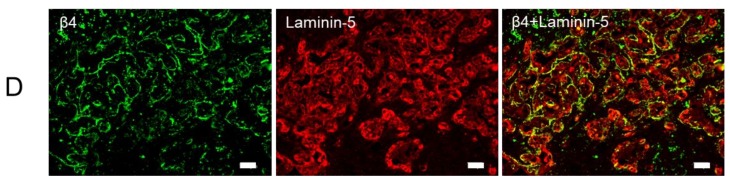
The relationship between β4 or β6 expression and laminin-5 or tenascin-C expression. Based on immunohistochemistry, the expression levels of β6 and tenascin-C are higher in intraductal-growth (IG) or periductal-infiltrating (PI) type intrahepatic cholangiocarcinomas than in the mass-forming (MF) type (**A**). β4 and laminin-5 expression levels are higher in non-peripheral central localization type than in the peripheral localization type (**B**). Based on immunofluorescence staining, β6 is located in the basal lamina, cell membrane, and cytoplasm (**C**, green). The intense expression of tenascin-C is shown in the cytoplasm of the tumor cells (**C**, red). The similar localization of β6 and tenascin-C is shown in intrahepatic cholangiocarcinoma. The tumor cells are confirmed by hematoxylin and eosin staining (**C**). Based on double immunofluorescence staining, the expression of β4 is located in the basal lamina, cell membrane, and cytoplasm (**D**, green). The dense expression of laminin-5 is shown in the cytoplasm of the tumor cells (**D**, red). In the merged images, there is coincident expression, but a different intracellular localization of β4 and laminin-5 are observed in intrahepatic cholangiocarcinoma (**D**, yellow). Scale bar, 50 μm.

The expressions of laminin-5, a ligand for β4, and tenascin-C, a ligand for β6, were observed in both the cytoplasm of tumor cells and the stroma within tumor tissues, but were predominantly observed within tumor cells. Laminin-5 and tenascin-C expressions were considered positive when more than 25% of the tumor cells or areas showed positive staining regardless of the intensity. Twenty-six and 18 cases were positive for laminin-5 and tenascin-C expressions, respectively. Therefore, β4 expression was significantly associated with laminin-5 expression within tumors (*p* < 0.001) (Table 1 and Figure 3B), but not in the invasive front portions of tumors, while β6 expression was significantly associated with tenascin-C expression in the tumor cells, or both tumor cells and the stroma (*p* = 0.016) (Table 1 and Figure 3A), but not in the stroma within tumor tissues alone. Furthermore, immunofluorescence staining was performed to confirm the relationship between the expressions of β4 and laminin-5, and β6 and tenascin-C, respectively. β6 was shown in the basal lamina, cell membrane, and cytoplasm, while tenascin-C was shown in the cytoplasm of the tumor cells. The expressions of β6 and tenascin-C were shown with similar localization within ICC (Figure 3C). β4 was shown in the basal lamina, cell membrane, and cytoplasm of the tumor cells, while laminin-5 was shown in the cytoplasm. In the merged images, the expressions of β4 and laminin-5 were observed at almost the same sites in ICC, but the coincident expression and different intracellular localizations of the two molecules was also apparent (Figure 3D). 

### 2.4. Relationships between Laminin-5 and Tenascin-C Expressions and Clinicopathological Features in Patients with ICC

Laminin-5 expression was significantly correlated with the macroscopic type, the growth type, hepatic vein invasion, and lymph node metastasis in ICC (Table 2). Laminin-5 expression levels were higher in the periductal-infiltrating or intraductal-growth types than in the mass-forming types (*p* = 0.019), and were also higher in the infiltrative-growth types than in the expansive-growth types (*p* = 0.045). High expression was related to hepatic vein invasion (*p* = 0.034) and lymph node metastasis (*p* = 0.029). Tenascin-C expression levels were significantly higher in the infiltrative-growth types than in the expansive growth types (*p* = 0.031) (Table 2).

**Figure 4:**
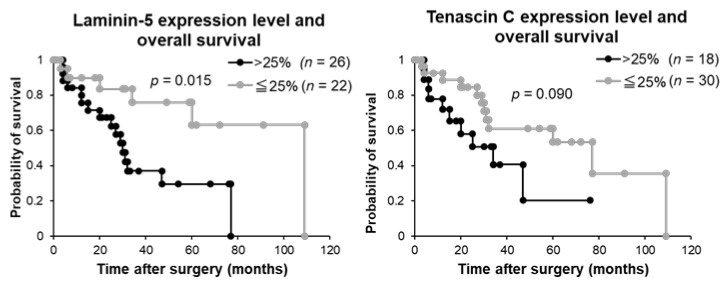
Laminin-5 and tenascin-C expression levels and overall survival analysis of patients with intrahepatic cholangiocarcinoma.

The expression level of laminin-5 was significantly correlated with OS (*p* = 0.015), while tenascin-C expression was not correlated (*p* = 0.090) (Figure 4).

### 2.5. Relationships between β6 Integrin Expression and TGF-β1 and α-SMA Expressions in ICC

**Figure 5:**
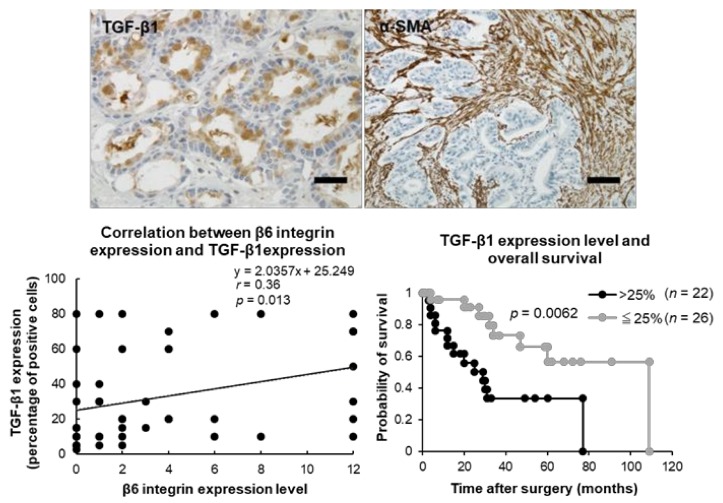
Expressions of transforming growth factor (TGF)-β1 and *α*-smooth muscle actin (SMA) in intrahepatic cholangiocarcinoma. TGF-β1 is shown in the cytoplasm of tumor cells, while *α*-SMA is shown in the stroma of intrahepatic cholangiocarcinoma. TGF-β1 expression is correlated with β6 integrin expression levels and overall survival rate. Scale bar, 50 μm.

TGF-β1 expression was observed in the cytoplasm of tumor cells and normal septal bile duct epithelium, while strong expression of *α*-SMA was observed in the stroma of ICC (Figure 5). TGF-β1 and *α*-SMA expressions were considered definitely positive when more than 25% of the tumor cells or stroma of the ICC showed positive staining. Twenty-two and 41 cases were positive for TGF-β1 and *α*-SMA expressions, respectively. β6 integrin expression was significantly correlated with TGF-β1 expression (*r* = 0.36, *p* = 0.013), but was not correlated with SMA (*r* = −0.040, *p* = 0.94). Moreover, TGF-β1 expression was associated with portal vein invasion (*p* = 0.022), intrahepatic metastasis (*p* = 0.049), and OS rate (*p* = 0.0062), but was not associated with lymph node metastasis (*p* = 0.31) in patients with ICC (Supplemental Table S2, Figure 5).

## 3. Discussion

In the present study, β4 and β6 integrin expression levels were higher in the non-peripheral central localization type ICC than in the peripheral localization type ICC. The levels were also higher in the periductal-infiltrating or intraductal-growth types than in the mass-forming types of ICC; and in the infiltrative-growth than in the expansive-growth type ICC. In addition, high expressions of β4 and β6 were associated with mucin production and the expression of cytoplasmic EMA. These results suggest that the subgroup of ICC with high integrin expression may partly resemble the large bile duct type defined by Aishima et al. [22], the bile duct type defined by Liau et al. [10], and the muc-ICC defined by Komuta et al. [9], as indicated by the non-peripheral central localization around the intrahepatic large bile ducts, periductal infiltration, and mucin production, as well as the cytoplasmic expression of EMA. However, further histopathological, immunohistochemical, and molecular evaluations are needed for concise comparative evaluations among these subclassifications. In our previous study of normal liver tissues, luminal EMA expression was observed in cholangioles, bile ductules, and peripheral small bile ducts, while cytoplasmic EMA expression was observed in larger bile ducts [21]. In addition, frequent expressions of β4 and β6 integrins were detected in the biliary epithelium of septal and larger bile ducts, while faint or no positive staining for both integrins was observed in the bile ductular epithelium of normal liver tissues [18]. These results indicated a close relationship between EMA immunolocalization or integrin expression and cholangiocyte phenotypes at different sites of the biliary tree. The subgroup of ICC with low or negative β4 and β6 integrin expression in the present study was characterized by peripheral localization, mass-forming gross types, poor mucin production, and luminal expression of EMA, indicating partial resemblance to the peripheral small bile duct type defined by Aishima et al. [22], the cholangiolar type identified by Liau et al. [10], and the mixed-ICC type described by Komuta et al. [9]. These results suggest that integrin expression is associated with ICC subclassification, as defined by the cholangiocyte phenotypes at different sites of the biliary tree.

Using an integrated genomic analysis, Sia et al. recently identified inflammation and proliferation types among 119 cases of ICC [23]. The proliferation type was characterized by the activation of the rat sarcoma viral oncogene homolog (*RAS*), *MAPK*, and *c-MET*, in which mutations in *KRAS* and *BRAF* resulted in a poor prognosis. However, the relationship between this molecular classification and previously described ICC subclassifications remains to be fully elucidated. In addition, genetic heterogeneity, including population level and intratumoral level, makes the identification of targeted genes and targeted therapy for ICC more complex [24,25]. Molecular or genetic analysis was not performed in the present study. In future studies, the relationship between integrin expression and molecular classification should be clarified.

The present study also showed that high β4 and β6 integrin expressions were related to the infiltrative growth and bile duct invasion of ICC. Additionally, β6-positive ICC, including the low expression group, was significantly associated with lymph node metastasis, indicating the close relationship between integrin expression and clinicopathological features in ICC, although integrin β4 and β6 expression levels were not significantly correlated with OS in the present study. The number of cases examined seems insufficient for survival analysis because metastasis, invasive growth, and histological grade are known as prognostic factors of ICC. In a study of α6β1 and α6β4, Ding et al. reported that α6 overexpression was significantly correlated with larger tumors, multiple nodules, microvascular/bile duct invasion, and lymphatic metastasis, and an lower OS rate in ICC patients; they indicated that high α6 expression enhanced the activation of extracellular signal-regulated kinases (ERK1/2) and protein kinase B (AKT) signals, inducing ICC cell metastasis and invasion [14]. Li et al. reported that β6 is up-regulated in cholangiocarcinoma, not only in ICC, and is also associated with tumor cell invasion and lymph node metastasis via a Rac1-dependent signal. Li et al. showed the diagnostic value of β6 overexpression, but did not show survival data [17]. Furthermore, in our previous study, *ITGB4* and *ITGB6* mRNA levels in cholangiocarcinoma cell lines were high in HuCCT1; however, almost no expression of either molecule was observed in HuH28, which is known to grow slowly [18,26]. Partly supporting our present data, these previous reports also indicated that integrin expression is associated with the biological behaviors of cholangiocarcinoma, including ICC, but not with tumor differentiation grade. The molecular mechanisms of their associations require investigation in future studies. In tumors other than ICC, β4 integrin is involved in the progression of breast, lung, colon, and prostate cancers [27,28,29,30]; β6 is up-regulated in various cancers, modulates tumor cell invasion, and may promote cancer progression and metastasis [31,32,33].

Laminin-5, a ligand of β4, is an ECM protein belonging to the laminin family that is mainly expressed in the basement membrane and has been reported in HCC as well as other cancers. Its expression is significantly correlated with cancer metastasis and poor prognosis [34]. Tenascin, a glycoprotein ligand of β6, is found in the ECM of normally developing embryonic tissues, and is involved in fetal development and oncogenesis, as well as in malignant tumor cell invasion and metastasis [35]. Aishima et al. reported the expression of laminin gamma 2 chain and tenascin, and demonstrated their prognostic significance in ICC [36,37]. In the present study, in particular, laminin-5 expression was significantly correlated with clinicopathological features of ICC including OS. We investigated the coincident expressions of β4 and its ligand, laminin-5, and β6 and its ligand, tenascin-C, in ICC, and demonstrated a significant association between integrin and its ligand expression in subgroups of ICC, suggesting a close interaction between integrin and its ligand in the biological behaviors of ICC.

TGF-β1 expression was correlated with β6 integrin expression as reported thus far. TGF-β1 is known to be activated by β6, stimulate myofibroblast proliferation, and promote fibrosis in cancer [38]. However, the expression of α-SMA was not correlated with β6 integrin expression in our present study. α-SMA positive stroma in ICCs was too abundant for a quantitative correlation analysis, because 85% of cases were definitely positive. An investigation of early fibrotic reactions may be required to evaluate the correlation between β6 integrin expression and cancer-associated α-SMA-positive myofibroblasts.

Taken together, the results of the present study showed, for the first time, the different levels of β4 and β6 integrin expression in ICC subgroups, and suggested that integrin expression is associated with the subclassification and clinicopathological features of ICC through the coincident expression of laminin-5 and tenascin-C, as well as of TGF-β1.

## 4. Materials and Methods

### 4.1. Patients and Tissue Samples

Tissue samples from 48 patients with ICC were obtained by surgical resection at Tokyo Medical and Dental University Hospital between 2007–2014. Tissue samples were fixed in a 10% formalin solution, and embedded in paraffin for histological diagnosis and immunohistochemistry analysis. The patients included 36 men and 12 women ranging from 39 to 84 years of age (mean, 70.5 years). Five patients were positive for the serum hepatitis B surface antigen (HBsAg), and seven were positive for the anti-hepatitis C virus (HCV) antibody; one was positive for both, and 35 were negative for both. The largest tumor diameters ranged from 18 mm to 220 mm (mean, 58.8 mm). CLCs and mixed cholangiohepatocellular carcinomas were excluded from the analysis. ICC was grossly classified into three groups: mass-forming (MF), periductal-infiltrating (PI), and intraductal-growth (IG), as well as mixed types (MF+PI and IG+PI). Based on surgical findings and macroscopic examination, the tumors were defined as peripheral localization types involving the septal and interlobular bile ducts, or as non-peripheral central localization types involving the first to third-order branches of the right and left hepatic bile ducts. The histological differentiation grades for ICC were assigned according to World Health Organization classifications [39]. The surrounding non-tumorous liver tissues showed normal liver in 30 patients, chronic hepatitis in 11 patients, and cirrhotic change in seven patients. The diagnosis of ICC was confirmed by immunohistochemical results that were positive for cytokeratin 7 and negative for hepatocyte marker (Hep Par-1), in addition to the histological findings.

Patient follow-up was completed on 31 March 2017. The median follow-up period was 32.2 months (range: 1–109 months). All of the patients that were followed at Tokyo Medical and Dental University Hospital were monitored postoperatively using chest-computed tomography (CT) and abdominal contrast-enhanced CT every 1 to 12 months, depending on the postoperative time. OS was defined as the period from surgery to death or the last observation point.

This study was approved by the ethics committee of Tokyo Medical and Dental University (No. M2000-2081; 8 May 2015).

### 4.2. Immunohistochemistry

Immunohistochemistry analysis was performed using monoclonal and polyclonal antibodies (Supplemental Table S3). Paraffin-embedded tissue sections that were 4 μm in thickness were deparaffinized with xylene and rehydrated with graded ethanol. Antigen retrieval was performed, as indicated in Supplemental Table S3. Endogenous peroxidase was quenched with 3% H_2_O_2_ in distilled water for 5 min. The slides were incubated with primary antibodies for 30 min at room temperature, followed by incubation with the secondary antibody and detection using an EnVision FLEX (Agilent Technologies, Santa Clara, CA, USA) according to the manufacturer’s protocol, and counterstained with hematoxylin.

### 4.3. Immunofluorescence Analysis

Immunofluorescence staining for β6 integrin and tenascin-C was performed on formalin-fixed, paraffin-embedded tissue sections (4 μm thick) of selected cases with representative histopathological types and immunohistochemical results. Tissue sections were blocked using the streptavidin/biotin blocking kit (Vector laboratories, Burlingame, CA, USA) for 15 min at room temperature, respectively, and incubated with anti-β6 antibody for 60 min at room temperature. The slides were washed with 0.1% Tween phosphate-buffered saline (PBS), and incubated with the biotinylated anti-mouse IgG (Vector). After being washed with 0.1% Tween PBS, the slides were incubated with the DyLight 488-labeled streptavidin (Vector) (green color), and diluted 1:100 in PBS for 5 min at room temperature. Furthermore, serial tissue sections of the same selected cases were stained for immunofluorescence analysis of tenascin-C, using an anti-tenascin antibody as the primary antibody, and DyLight 549-labeled streptavidin (Vector) for red immunofluorescence detection.

For double immunofluorescence of β4 integrin and laminin-5, rabbit polyclonal anti-β4 antibody was used as the primary antibody, and biotinylated anti-rabbit IgG (Vector) was used as the secondary antibody in the first layer of the staining with green DyLight 488-labeling, and mouse monoclonal anti-laminin-5 antibody was used as the primary antibody and biotinylated anti-mouse IgG (Vector) was used as the secondary antibody in the second layer with red DyLight 549-labeling; these were then applied on the same tissue sections. The fixation, antigen retrieval, blocking, and washing steps were conducted as described above. Immunofluorescence was visualized using the Keyence BZ-X710 microscope (Keyence, Osaka, Japan).

### 4.4. Evaluation of Immunohistochemical Staining

The intensity of β4 and β6 integrin expressions was semi-quantitatively scored as 0 (negative), 1 (weak), 2 (moderate), or 3 (strong). The percentages of cells or areas positive for β4 or β6 were semi-quantitatively scored into five categories: 0 (<1%), 1 (1–25%), 2 (26–50%), 3 (51–75%), or 4 (76–100%). The relationships between β4 or β6 integrin expressions and clinicopathological parameters were evaluated by using a final score, which was calculated by multiplying the scores of staining intensities by the scores for the percentages of positive cells. Based on the final score, the cases were grouped as negative (a score of 0), low (1–4), and high (5–12). Mucin production was assessed by using Alcian blue staining (pH 2.5), and defined as positive for >10% positivity within glandular lumens or cytoplasm. Epithelial membrane antigen (EMA) expression patterns were categorized as dominantly luminal or cytoplasmic in the positive areas. The percentages of positive cells and stromal areas for laminin-5, tenascin-C, TGF-β1, and α-SMA were scored into five categories: 0 (<1%), 1 (1–25%), 2 (26–50%), 3 (51–75%), and 4 (76–100%). Negative was defined as category 0, focally positive was defined as category 1, and definitely positive was defined as >25% (more than two in the category) of tumor cells or areas positive for cytoplasmic and stromal staining.

### 4.5. Statistical Analysis

The correlations among the clinicopathological findings; mucin production; EMA, laminin-5 and tenascin-C expressions; β4 and β6 integrin expression, and laminin-5, tenascin-C, and TGF-β1 expression in ICC were assessed using Fisher’s exact or *χ*^2^ and Student’s *t*-tests. Survival analysis based on the Kaplan–Meier method and log-rank tests were used to assess the survival data. The correlations between TGF-β1 and α-SMA expressions and β6 integrin expression in ICC were analyzed based on Spearman’s rank correlation coefficient. Data are presented as means ± standard deviations. *p* values < 0.05 were considered significant.

## 5. Conclusions

The present study showed, for the first time, the different levels of β4 and β6 integrin expression in ICC subgroups, and suggested that integrin expression is associated with the subclassification and clinicopathological features of ICC through the coincident expression of laminin-5 and tenascin-C, as well as of TGF-β1.

## Figures and Tables

**Table 1:** Correlation between β4 and β6 expressions and clinicopathological characteristics in intrahepatic cholangiocarcinoma.

		Number of Cases (*n* = 48)	β4 integrin Expression			β6 integrin Expression		
			Negative-Low *(n* = 29)	High (*n* = 19)	*p*-Value	Negative-Low (*n* = 36)	High (*n* = 12)	*p*-Value
Gender	Male	36	23	13	0.39	26	10	0.36
	Female	12	6	6		10	2	
Age (mean) (years)			71.5 (51–84)	69.1 (39–84)	0.42	70.4 (39–84)	71.1 (51–82)	0.83
Tumor size (mean) (mm)			60.2 (20–220)	56.7 (18–140)	0.78	61.9 (18–220)	49.4 (23–100)	0.20
Localization	Peripheral	38	26	12	0.033 *	32	6	0.0092 **
	Non-peripheral	10	3	7		4	6	
Macroscopic type	MF	42	29	13	0.0022 ***	34	8	0.028 *
	MF+PI, IG+PI, PI	6	0	6		2	4	
Histological differentiation	Well	6	3	3	0.023 *	2	4	0.0068 **
	Moderate	29	14	15		21	8	
	Poor	13	12	1		13	0	
Growth type	Expansive	23	18	5	0.015 *	20	3	0.065
	Infiltrative	25	11	14		16	9	
HBV	+	6	5	1	0.22	6	0	0.15
	−	42	24	18		30	12	
HCV	+	8	6	2	0.31	6	2	0.68
	−	40	23	17		30	10	
Cirrhosis	+	7	4	3	0.58	7	0	0.11
	−	41	25	16		29	12	
Serosa invasion	+	24	12	12	0.14	16	8	0.18
	−	24	17	7		20	4	
Portal vein invasion	+	39	23	16	0.49	27	12	0.056
	−	9	6	3		9	0	
Hepatic vein invasion	+	21	10	11	0.11	14	7	0.24
	−	27	19	8		22	5	
Hepatic artery invasion	+	4	2	2	0.52	3	1	0.74
	−	44	27	17		33	11	
Bile duct invasion	+	26	11	15	0.0053 **	15	11	0.0026 ***
	−	22	18	4		21	1	
Intrahepatic metastasis	+	21	10	11	0.11	13	8	0.065
	−	27	19	8		23	4	
Lymph node metastasis	+	14	6	8	0.11	9	5	0.93
	−	34	23	11		27	7	
Alcian blue stain	≤10%	12	11	1	0.011 *	12	0	0.018 *
	>10%	36	18	18		24	12	
EMA	Luminal pattern	11	10	1	0.018 *	10	1	0.16
	Cytoplasmic pattern	37	19	18		26	11	
Laminin-5	≤25%	22	21	1	<0.001 ***	n.d.	n.d.	
	>25%	26	8	18		n.d.	n.d.	
Tenascin-C	≤25%	30	n.d.	n.d.		26	4	0.016 *
	>25%	18	n.d.	n.d.		10	8	

MF: mass-forming type, PI: periductal-infiltrating type, IG: intraductal-growth type, HBV: hepatitis B virus, HCV: hepatitis C virus, EMA: epithelial membrane antigen, n.d.: no data; +: positive; −: negative; * *p* < 0.05; ** *p* < 0.01; *** *p* < 0.005.

**Table 2:** Correlation between laminin-5 and tenascin-C expressions and clinicopathological characteristics in intrahepatic cholangiocarcinoma.

		Number of Cases (*n* = 48)	Laminin-5 Expression			Tenascin-C Expression		
			≤25% (*n* = 22)	>25% (*n* = 26)	*p*-Value	≤25% (*n* = 30)	>25% (*n* = 18)	*p*-Value
Gender	Male	36	17	19	0.74	21	15	0.25
	Female	12	5	7		9	3	
Age (mean) (years)			72.5 (59–84)	68.9 (39–84)	0.21	72.5 (53–84)	67.3 (39–83)	0.084
Tumor size (mean) (mm)			64.6 (20–220)	53.9 (18–140)	0.37	56.9 (20–150)	61.9 (18–220)	0.68
Localization	Peripheral	38	18	20	0.48	25	13	0.29
	Non-peripheral	10	4	6		5	5	
Macroscopic type	MF	42	22	20	0.019 *	27	15	0.40
	MF+PI, IG+PI, PI	6	0	6		3	3	
Histological differentiation	Well	6	3	3	0.74	3	3	0.15
	Moderate	29	12	17		16	13	
	Poor	13	7	6		11	2	
Growth type	Expansive	23	14	9	0.045 *	18	5	0.031 *
	Infiltrative	25	8	17		12	13	
Serosa invasion	+	24	10	14	0.56	14	10	0.55
	−	24	12	12		16	8	
Portal vein invasion	+	39	18	21	0.61	24	15	0.55
	−	9	4	5		6	3	
Hepatic vein invasion	+	21	6	15	0.034 *	12	9	0.50
	−	27	16	11		18	9	
Hepatic artery invasion	+	4	2	2	0.63	2	2	0.48
	−	44	20	24		28	16	
Bile duct invasion	+	26	10	16	0.27	14	12	0.18
	−	22	12	10		16	6	
Intrahepatic metastasis	+	21	7	14	0.59	11	10	0.20
	−	27	15	22		19	8	
Lymph node metastasis	+	14	3	11	0.029 *	7	7	0.21
	−	34	19	15		23	11	

MF: mass-forming type, PI: periductal-infiltrating type, IG: intraductal-growth type; +: positive; −: negative; * *p* < 0.05.

## Supplementary Materials

Supplementary materials can be found at http://www.mdpi.com/1422-0067/19/4/1004/s1.
