# Supplementary material for: β4 and β6 Integrin Expression Is Associated with the Subclassification and Clinicopathological Features of Intrahepatic Cholangiocarcinoma
Source: Int J Mol Sci. 2018 Mar 27;19(4):1004. doi: 10.3390/ijms19041004 (PMC5979350; doi:10.3390/ijms19041004)
Supplement: Supplementary file 1 [file ijms-19-01004-s001.zip › ijms-282765-SI/Supplement Table S1.pdf]

Supplemental Table S1. Relationship between β6 integrin expression and clinicopathological characteristics of intrahepatic cholangiocarcinoma.

		Number of cases	β6 integrin expression		
		(n = 48)	Negative (n = 13)	Positive (n = 35)	*p*-Value
Gender	Male	36	11	25	0.29
	Female	12	2	10	
Age (mean) (years)			73.7 (53-84)	69.4 (39-84)	0.26
Tumor size (mean) (mm)			60.2 (20-220)	58.3 (18-150)	0.88
Localization	Peripheral	38	13	25	0.028*
	Non-peripheral	10	0	10	
Macroscopic type	MF	42	13	29	0.13
	MF+PI, IG+PI, PI	6	0	6	
Histological differentiation	Well	6	1	5	0.51
	Moderate	29	7	22	
	Poor	13	5	8	
Growth type	Expansive	23	9	14	0.069
	Infiltrative	25	4	21	
Serosa invasion	+	24	4	20	0.096
	-	24	9	15	
Portal vein invasion	+	39	9	30	0.18
	-	9	4	5	
Hepatic vein invasion	+	21	3	18	0.074
	-	27	10	17	
Hepatic artery invasion	+	4	1	3	0.71
	-	44	12	32	
Bile duct invasion	+	26	4	22	0.048*
	-	22	9	13	
Intrahepatic metastasis	+	21	4	17	0.21
	-	27	9	18	
Lymph node metastasis	+	14	1	13	0.045*
	-	34	12	22	

MF: mass-forming type, PI: periductal-infiltrating type, IG: intraductal-growth type; *, $p < 0.05$
