# Supplementary material for: β4 and β6 Integrin Expression Is Associated with the Subclassification and Clinicopathological Features of Intrahepatic Cholangiocarcinoma
Source: Int J Mol Sci. 2018 Mar 27;19(4):1004. doi: 10.3390/ijms19041004 (PMC5979350; doi:10.3390/ijms19041004)
Supplement: Supplementary file 1 [file ijms-19-01004-s001.zip › ijms-282765-SI/Supplement Table S2.pdf]

Supplemental Table S2. Relationship between transforming growth factor (TGF)-β1 expression and clinicopathological characteristics of intrahepatic cholangiocarcinoma.

		Number of cases (n = 48)	TGF-β1 expression		
			≦25% (n = 26)	>25% (n = 22)	*p*-Value
Gender	Male	36	17	19	0.09
	Female	12	9	3	
Age (mean) (years)			71.9 (53-84)	69.0 (39-84)	0.33
Tumor size (mean) (mm)			50.7 (18-110)	68.4 (20-220)	0.16
Localization	Peripheral	38	22	16	0.26
	Non-peripheral	10	4	6	
Macroscopic type	MF	42	24	18	0.26
	MF+PI, IG+PI, PI	6	2	4	
Histological differentiation	Well	6	5	1	0.08
	Moderate	29	12	17	
	Poor	13	9	4	
Growth type	Expansive	23	12	11	0.79
	Infiltrative	25	14	11	
Serosa invasion	+	24	11	13	0.25
	-	24	15	9	
Portal vein invasion	+	39	18	21	0.022*
	-	9	8	1	
Hepatic vein invasion	+	21	10	11	0.42
	-	27	16	11	
Hepatic artery invasion	+	4	2	2	0.63
	-	44	24	20	
Bile duct invasion	+	26	12	14	0.23
	-	22	14	8	
Intrahepatic metastasis	+	21	8	13	0.049*
	-	27	18	9	
Lymph node metastasis	+	14	6	8	0.31
	-	34	20	14	

MF: mass-forming type, PI: periductal-infiltrating type, IG: intraductal-growth type; *, $p < 0.05$
