# Supplementary material for: β4 and β6 Integrin Expression Is Associated with the Subclassification and Clinicopathological Features of Intrahepatic Cholangiocarcinoma
Source: Int J Mol Sci. 2018 Mar 27;19(4):1004. doi: 10.3390/ijms19041004 (PMC5979350; doi:10.3390/ijms19041004)
Supplement: Supplementary file 1 [file ijms-19-01004-s001.zip › ijms-282765-SI/Supplement Table S3.pdf]

Supplemental Table S3. Primary antibodies and antigen retrieval methods.

Antibody	Clone	Dilution	Antigen retrieval	Source
Rabbit polyclonal anti-β4 integrin		1:200	pH9, 95℃, 40min	Santa Cruz
Mouse monoclonal anti-β6 integrin	442.5C4	1:400	Proteinase K, 10min	Calbiochem
Mouse monoclonal anti-laminin-5	4G1	1:50	pH9, 95℃, 40min	Agilent
Mouse monoclonal anti-tenascin-C	E-9	1:50	pH9, 121℃, 15min	Santa Cruz
Mouse monoclonal anti-cytokeratin 7	OV-TL12/30	1:60	pH9, 95℃, 40min	Agilent
Mouse monoclonal anti-Hep Par-1	OCH1E5	1:60	pH6, 95℃, 40min	Agilent
Mouse monoclonal anti-EMA	E29	1:200	pH6, 95℃, 40min	Agilent
Rabbit polyclonal anti-TGF-beta 1		1:200	pH9, 95℃, 40min	Protein tech
Mouse monoclonal anti-alpha-SMA	1A4	1:100	pH6, 95℃, 20min	Agilent

EMA: epithelial membrane antigen, SMA: smooth muscle actin
