# Supplementary figures and images for: β4 and β6 Integrin Expression Is Associated with the Subclassification and Clinicopathological Features of Intrahepatic Cholangiocarcinoma
Source: Int J Mol Sci. 2018 Mar 27;19(4):1004. doi: 10.3390/ijms19041004 (PMC5979350; doi:10.3390/ijms19041004)

**Supplemental Figure S1.**

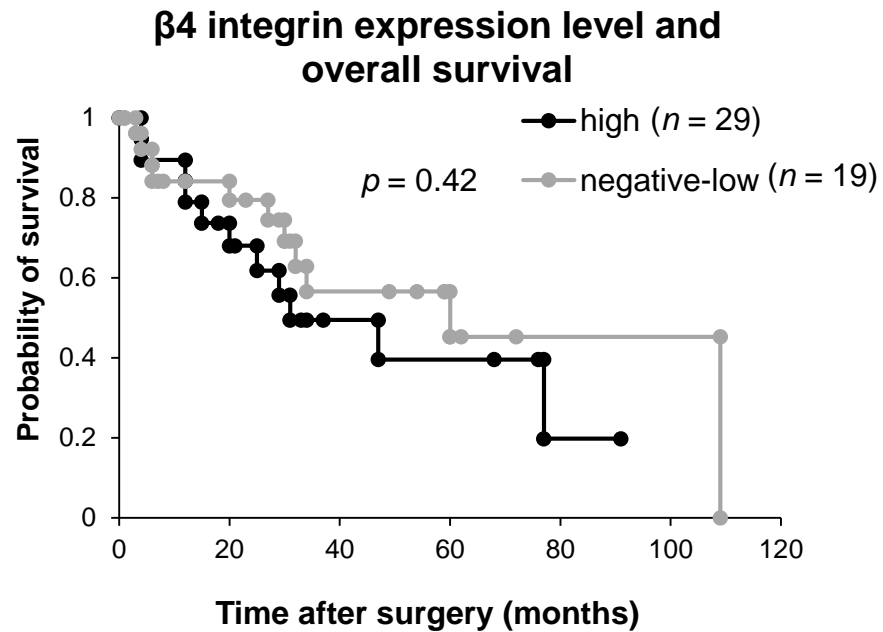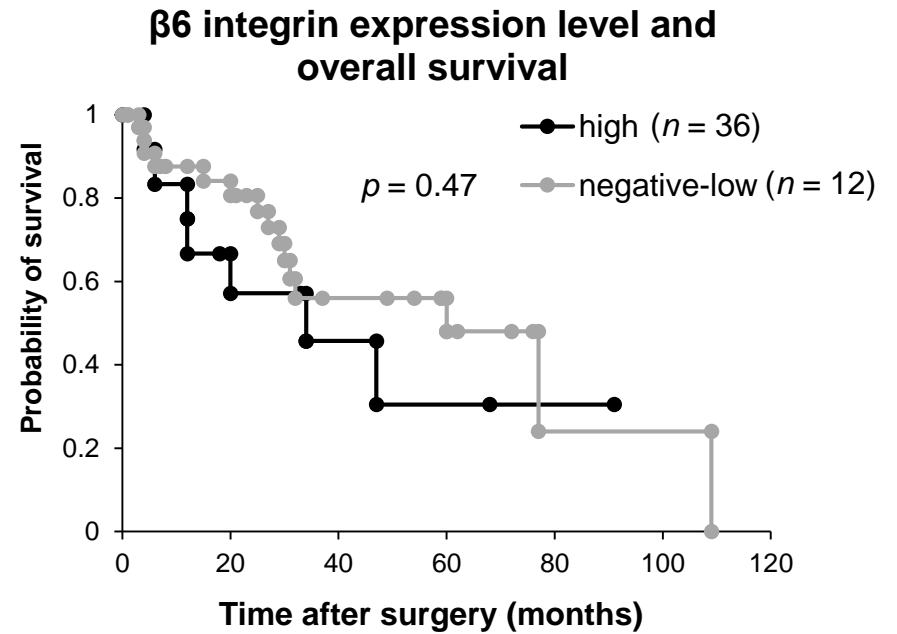

Supplement: Supplementary file 1 [file ijms-19-01004-s001.zip › ijms-282765-SI/Supplemental figure.pdf]
